# The promotive effect of ocean literacy on marine conservation behavior: A qualitative study based on Chinese university students

**DOI:** 10.1371/journal.pone.0323510

**Published:** 2025-08-08

**Authors:** Linzhao Wang, Bo Gao, Xiangwei Chang, Li Zhang

**Affiliations:** 1 Mental Health Education and Counseling Center, Guangdong Ocean University, Zhanjiang, Guangdong, China; 2 College of Literature and Journalism, Guangdong Ocean University, Zhanjiang, Guangdong, China; 3 College Students’ Mental Health Education Center, Henan Polytechnic Institute, Nanyang, Henan, China; 4 College of Food Science and Technology, Guangdong Ocean University, Zhanjiang, Guangdong, China; University of Mpumalanga, SOUTH AFRICA

## Abstract

Ocean literacy plays a crucial role in promoting sustainable marine conservation behaviors, yet its underlying psychological mechanisms among university students remain underexplored, particularly within Asian contexts. This qualitative study aimed to investigate how ocean literacy influences pro-environmental behaviors among Chinese university students through environmental beliefs, values, and perceived responsibility. Semi-structured interviews with 16 university students from four coastal universities in China were conducted and analyzed using thematic analysis with NVivo software. Results indicated that ocean literacy encompasses five interconnected dimensions: scientific knowledge, environmental ethics, behavioral intentions for conservation, policy understanding, and critical thinking. Emotional connectedness and direct experiential engagement emerged as significant factors enhancing students’ environmental awareness and behavioral transformation. The findings also highlighted cultural relevance, social norms, and interactive educational practices as essential elements for translating ocean literacy into tangible conservation actions. Despite participants demonstrating foundational ocean knowledge, limitations in their systemic understanding of complex marine issues were identified, suggesting the need for more comprehensive educational interventions. This study provides empirical insights for optimizing marine education strategies and policy initiatives aimed at enhancing public engagement and achieving sustainable ocean governance.

## Introduction

As the Earth’s largest life-support system, the ocean functions not only as a reservoir of global biodiversity but also as a cornerstone for climate regulation and the sustainable development of human societies. Its complex ecosystems—including coral reefs, seagrass beds, and mangroves—deliver irreplaceable ecosystem services such as carbon sequestration, oxygen production, nutrient cycling, and habitats for millions of species [1,2]. However, anthropogenic activities—such as overfishing, pollution, plastic waste accumulation, ocean acidification, and habitat destruction—have increasingly compromised the resilience of marine ecosystems, posing serious threats to ecological balance and global sustainability [3,4].

In response to this escalating ecological crisis, global environmental governance has elevated ocean conservation as a key international priority. Both the United Nations (UN) and the World Health Organization (WHO) underscore the vital role of interdisciplinary collaboration, public education, and behavioral interventions in fostering emotional connections between humans and the natural world, thereby advancing the Sustainable Development Goals (SDGs) [5]. Global initiatives such as the “United Nations Decade of Ocean Science for Sustainable Development” (2021–2030) call for capacity building, technological innovation, and educational cooperation to safeguard ocean health on a global scale [6]. Simultaneously, international frameworks such as the United Nations Convention on the Law of the Sea (UNCLOS) and the Convention on Biological Diversity (CBD) explicitly define state responsibilities in marine governance and mandate cross-border knowledge sharing.

Marine education — particularly the concept of Ocean Literacy (OL), which emphasizes public awareness, a sense of responsibility, and behavioral change—has been widely recognized as a key strategy for enhancing environmental consciousness and promoting sustainable marine management. The term OL was first introduced by Cava, who highlighted the importance of understanding the interactions between the ocean and human society, as well as the ability to make informed and responsible decisions [7]. Fauville further expanded this definition, conceptualizing OL as a multidimensional construct encompassing knowledge, attitudes, emotional engagement, and behavioral intentions, thereby framing it as a comprehensive literacy framework [8]. Recent studies increasingly underscore the significance of emotional engagement, value orientation, and systems thinking in shaping effective ocean literacy [9,10].

Enhancing ocean literacy among young people, particularly university students — has been demonstrated to be an effective means of fostering pro-environmental behavior. Research shows that students with higher levels of OL are more likely to engage in environmentally responsible practices, such as participating in beach clean-ups and reducing plastic consumption [11,12]. However, the relationship between ocean literacy and behavioral change is not straightforward; it is deeply shaped by psychological mechanisms such as environmental beliefs, value systems, and a sense of personal responsibility [13,14]. In recent years, growing scholarly attention has focused on identifying these “psychological pathways” that mediate the transformation of ocean literacy into sustainable actions [15,16].

While ocean literacy research in Western contexts is relatively well developed, significant gaps remain in studies targeting Asian populations—particularly university students in China. Longo and Chang note that Asian students often demonstrate a disconnect between environmental knowledge and actual behavior, highlighting the urgent need to develop culturally responsive and context-sensitive educational frameworks [17,18]. With more than 18,000 kilometers of coastline, China faces some of the world’s most severe challenges related to marine pollution and ecological degradation. Therefore, advancing ocean literacy among Chinese university students is not only an educational priority but also a crucial aspect of national ecological governance. Structured educational interventions combined with increased opportunities for public engagement are essential for fostering emotional attachment to the ocean, reinforcing environmental responsibility, and promoting sustainable behaviors.

This study employs a qualitative research design focusing on Chinese university students to explore how ocean literacy facilitates pro-environmental behavior through internal psychological mechanisms such as environmental beliefs, values, and responsibility. Using semi-structured interviews and thematic analysis, this research aims to reveal how individuals—shaped by multidimensional factors including knowledge, emotions, and sociocultural contexts—develop and transform ocean literacy into concrete sustainable actions. The findings are intended to inform the design of more targeted educational strategies and policy interventions that support both theoretical and practical advancements in ocean sustainability governance.

## Theoretical framework

Ocean literacy (OL) is a multidimensional and interdisciplinary construct grounded in key theories from environmental psychology, education, and sustainability science. According to Cava et al., individuals with a high level of ocean literacy should demonstrate three essential competencies: (1) a foundational understanding of ocean science, (2) the ability to effectively communicate ocean-related information, and (3) the capacity to make informed and responsible decisions regarding issues related to ocean resource management [7,8]. The European Marine Board further emphasizes that ocean literacy goes beyond cognitive understanding; it also requires individuals to make reasoned judgments about the complex relationships between humans and the marine environment and to engage in sustainable actions at both personal and societal levels [19]. In recent years, the definition of ocean literacy has evolved from a primarily knowledge-based framework to a more integrative model that encompasses cognitive, emotional, and behavioral dimensions [3,20]. Notably, McKinley et al. emphasize that “systems thinking” and “emotional closeness” are critical components for translating ocean knowledge into sustainable behaviors [20].

The theoretical foundation of this study integrates three well-established psychological frameworks: the Theory of Planned Behavior (TPB), the Value-Belief-Norm theory (VBN), and the Norm Activation Model (NAM). TPB posits that attitudes, subjective norms, and perceived behavioral control are key predictors of behavioral intentions and actions [14]. Within the context of ocean literacy, TPB suggests that extensive marine knowledge and positive attitudes toward marine conservation significantly influence an individual’s intention to act, thereby shaping pro-environmental behavior. The VBN theory asserts that biospheric values and environmental beliefs activate personal moral norms and a sense of obligation, which in turn lead to environmentally responsible behavior [13]. Specifically, individuals who endorse strong biospheric or altruistic values are more likely to develop heightened environmental awareness and a perceived sense of responsibility, motivating engagement in conservation actions. The NAM framework, meanwhile, highlights the central role of moral obligation and environmental responsibility in activating pro-environmental behavior [21]. Thus, ocean literacy may serve as a psychological catalyst, enhancing awareness of ocean crises and reinforcing personal responsibility, ultimately invoking moral imperatives to protect the marine environment. This study further explores how Chinese students’ behavioral transformations are influenced by geographic context, cultural identity, and prevailing social norms.

Additionally, qualitative research methods provide a valuable opportunity to examine individuals lived experiences, beliefs, and emotional connections to the marine environment. Through the use of semi-structured interviews and thematic analysis, this study aims to uncover nuanced insights into how ocean literacy shapes the attitudes and behaviors of Chinese university students. Qualitative methods are particularly well-suited to capturing the complexity and culturally embedded meanings of environmental behavior—perspectives that may be overlooked by purely quantitative approaches [22,23].

## Research status and hypothesis development

Recent advances in ocean literacy research have broadened the concept from a purely knowledge-based model to a more comprehensive framework that integrates attitudes, behaviors, emotional connections, and personal engagement with ocean-related issues [3,20]. Brennan et al. underscore the multidimensional nature of OL, identifying key components such as knowledge, awareness, attitudes, communication, behavior, and activism—all of which directly contribute to effective marine conservation efforts [3]. Similarly, McKinley et al. emphasize the importance of understanding the human–ocean relationship in shaping sustainable marine behaviors [20].

In Asia, ocean literacy research remains in a formative stage, often characterized by limited knowledge levels and insufficient behavioral transformation [24]. Existing studies, which primarily focus on university students, suggest that pro-environmental attitudes are significantly influenced by participation in marine-related courses and individual characteristics. Students with higher levels of OL are more likely to adopt environmentally friendly behaviors [25,26]. In recent years, the digital transformation accelerated by the post-pandemic era has created new opportunities for ocean literacy education and the promotion of pro-environmental behaviors. Emerging evidence indicates that virtual reality (VR), blended learning, and online collaborative platforms significantly enhance students’ systems thinking and environmental engagement [27,28]. Digital innovation not only expands access to learning but also deepens students’ emotional connections to the ocean and strengthens their sense of social responsibility [20]. Thus, digital technologies have become pivotal drivers for advancing both ocean literacy and sustainable behaviors.

The relationship between ocean literacy and pro-environmental behavior is well documented. Ashley et al. argue that improved ocean literacy is positively correlated with proactive marine conservation actions, as increased literacy heightens awareness and supports responsible decision-making [12]. Existing evidence also affirms a significant association between university students’ OL levels and their concern for the marine environment. National surveys in countries such as Canada reveal that although students’ scientific knowledge of the ocean varies, those with higher levels of OL tend to express stronger interest in marine environmental protection and sustainable resource management [29,30]. This correlation is not limited to student populations; similar patterns have been observed across various age groups, regions, and educational backgrounds [31]. Furthermore, studies suggest that enhanced OL strengthens key psychological factors that support pro-ocean behavior [10]. Although ocean literacy does not always directly lead to behavioral change, it is often mediated by individual environmental beliefs, perceived social norms, and perceived behavioral control [14,32].

Based on the existing literature and theoretical frameworks, this qualitative study aims to explore the following hypotheses concerning Chinese university students:

H1: Students articulate the relationship between ocean literacy and pro-environmental behavior primarily through personal experiences and perceived social influences.

H2: Students’ ocean-related values positively correlate with their awareness and comprehension of both global and regional marine environmental issues.

H3: Educational interventions significantly enhance students’ sense of environmental responsibility and their willingness to engage in marine conservation behaviors.

H4: Higher ocean literacy levels enable students to better articulate the importance of ocean-related beliefs, values, and responsibilities in shaping their pro-environmental behaviors.

H5: Cultural, social, and contextual factors substantially influence students’ ability to translate ocean literacy into concrete pro-environmental actions.

Through the exploration of these hypotheses, this study aims to deepen the understanding of the complex relationships among ocean literacy, environmental beliefs, values, responsibility, and pro-environmental behaviors in the context of Chinese university students. The findings are expected to provide a strong empirical and theoretical foundation for the development of targeted educational strategies and policy interventions in the field of marine sustainability.

## Materials and methods

### Research design

This study aimed to investigate the levels of ocean literacy among Chinese university students and examine its relationship with pro-ocean behavior. A qualitative research design was adopted, with semi-structured interviews serving as the primary data collection method to generate rich and in-depth insights. The interviews were carefully structured to address multiple dimensions, including ocean knowledge, environmental beliefs, value orientations, sense of responsibility, and actual environmental behaviors. The goal was to develop a comprehensive understanding of how university students perceive marine environmental issues and how such awareness is transformed into tangible conservation practices. This qualitative approach was intended to uncover the underlying psychological mechanisms that link ocean literacy to pro-environmental behavior.

### Data analysis tool

Data were analyzed using NVivo 12, a qualitative data analysis software developed by QSR International (Australia). Widely adopted in academic research, NVivo provides functions for coding, categorizing, and thematically analyzing qualitative data, enabling the effective organization, analysis, and synthesis of unstructured data such as interview transcripts, open-ended questionnaires, and relevant literature [22]. The software also supports node construction based on Boolean logic and the development of conceptual maps, allowing researchers to identify key themes and explore the interrelationships among them within the interview data. These analytical capabilities facilitated a systematic examination of the psychological characteristics and underlying mechanisms that influence Chinese university students’ ocean literacy and their engagement in pro-ocean behaviors [33].

### Participant selection

To explore Chinese university students’ perceptions and conceptions of ocean literacy in greater depth, this study recruited 16 students from four universities located in eastern (Jiangsu Ocean University and Zhejiang Ocean University) and southern China (Guangdong Ocean University and Guangxi University) through voluntary participation. The participants had varying levels of interest in or practical experience with marine environmental issues. One-on-one semi-structured interviews were conducted face-to-face, each lasting approximately 20–44 minutes to ensure that participants could fully articulate their views. To enhance the diversity and representativeness of the sample, participants varied in demographic characteristics such as gender, age, and academic background. The sample included eight female and eight male students, aged between 19 and 23, with academic majors spanning the humanities, sciences, and engineering. To ensure confidentiality, all participants were anonymized using codes A1 to A16. Detailed demographic information is presented in Table 1.

**Table 1:** Characteristics of the interview participants (N = 16).

No.	Gender	Province	Major	Age	Interview time (minutes)
A1	Female	Guangdong Province	Liberal arts	20	44.08
A2	Female	Guangdong Province	Liberal arts	19	25.57
A3	Female	Zhejiang Province	Liberal arts	20	29.02
A4	Female	Guangdong Province	Sciences	23	32.20
A5	Female	Guangdong Province	Engineering	21	34.13
A6	Female	Guangdong Province	Sciences	22	25.26
A7	Male	Zhejiang Province	Sciences	22	27.26
A8	Female	Guangxi Province	Engineering	19	20.27
A9	Female	Jiangsu Province	Engineering	19	33.14
A10	Male	Jiangsu Province	Sciences	21	32.21
A11	Male	Guangdong Province	Engineering	21	23.01
A12	Male	Guangdong Province	Engineering	21	20.19
A13	Male	Guangxi Province	Sciences	21	24.02
A14	Male	Guangdong Province	Engineering	21	24.49
A15	Male	Guangdong Province	Sciences	22	22.14
A16	Male	Guangxi Province	Sciences	22	26.46

### Data collection

Data was collected in July 2024 through individual semi-structured interviews, each lasting approximately 30–60 minutes. The interview protocol focused on multiple aspects, including students’ ocean literacy, cognitive understanding of marine issues, perceptions of the ocean’s influence on their personal development, the formation of marine environmental beliefs, and how these beliefs inform daily decision-making and pro-ocean behaviors. All interviews were audio-recorded with the participants’ consent and transcribed verbatim using professional transcription software. The final dataset comprised a total of 116,962 words, with individual transcripts ranging from 4,685–12,426 words. All transcripts were anonymized, and participants were identified using numerical codes to protect their privacy.

This study strictly adhered to ethical research standards and received approval from the Institutional Ethics Review Board (Approval No.: 1041386–202407-HR-106–02). All participants were fully informed of the study’s objectives and voluntarily participated after signing a written informed consent form. Anonymity and confidentiality were ensured throughout the research process in accordance with the established ethical guidelines.

### Research team and coding procedures

Given that the coding process in qualitative research inherently involves researchers’ subjective interpretation of textual data, this study adopted multiple strategies to ensure analytical rigor and objectivity. A four-member expert panel was convened, consisting of two Ph.D. holders in educational psychology and two university faculty members, all with prior experience in qualitative research and a strong understanding of the theoretical foundations relevant to this study. The primary coding tasks were independently performed by two members of the research team. Data analysis followed the grounded theory approach [34], proceeding through the following structured phases:

(1) Open Coding:

The two coders independently reviewed and analyzed all interview transcripts line by line, identifying key words, phrases, or statements closely aligned with the research objectives. These were assigned as initial open codes. For example, the statement “Protecting the ocean is everyone’s responsibility” was coded under the node “sense of responsibility.” After completing their independent coding, the two researchers compared results, documented discrepancies, and engaged in discussion to resolve inconsistencies. Code definitions were refined with reference to relevant literature and theoretical frameworks, resulting in a preliminary set of unified codes.

(2) Axial Coding and Node Categorization:

The open codes were subsequently organized into broader, conceptually related categories, forming axial codes or tree-structured nodes. For instance, codes such as “sense of responsibility,” “environmental awareness,” and “behavioral motivation” were clustered under the broader theme of “environmental responsibility consciousness.” When discrepancies in categorization emerged, the researchers engaged in iterative discussions to reach consensus. In cases where agreement could not be achieved, unresolved issues were submitted to the full research panel for group deliberation until a consensus on final categorization was reached.

(3) Selective Coding and Theoretical Framework Development:

Building on the axial coding stage, the researchers advanced to selective coding, identifying core themes and conceptualizing the overarching theoretical framework. This phase aimed to abstract central relationships—such as the internal mechanisms linking ocean literacy to pro-ocean behavior—and structure the framework of the study. The selective coding was independently conducted by the two researchers, followed by comparative review and in-depth discussion to ensure thematic clarity, coherence, and consistency.

Following the preliminary coding phases, the full expert team reviewed the node structure collaboratively. When necessary, nodes were merged, redefined, or adjusted to ensure definitional clarity and logical alignment. Additionally, demographic variables such as gender, age, academic major, and environmental attitudes were incorporated into case nodes to enhance the interpretive depth and contextual richness of the dataset. This systematic and transparent coding protocol enhanced the analytical rigor and ensured the credibility and trustworthiness of the qualitative findings.

## Results

### Text processing and word frequency analysis

To ensure the systematic and timely analysis of qualitative data, this study adopted a “concurrent interview–transcription–preliminary analysis” strategy. Following each round of interviews, transcription, note-taking, and initial coding were conducted immediately. The interview protocol was dynamically adjusted based on emerging insights, thereby enhancing analytical sensitivity and facilitating the achievement of theoretical saturation. Audio recordings were transcribed using an AI-assisted transcription platform, after which the research team meticulously reviewed and revised the transcripts segment by segment to ensure semantic accuracy. The final corpus comprised 116,962 Chinese characters and was imported into NVivo 12 for data management, coding, and word frequency analysis.

**Fig 1:**
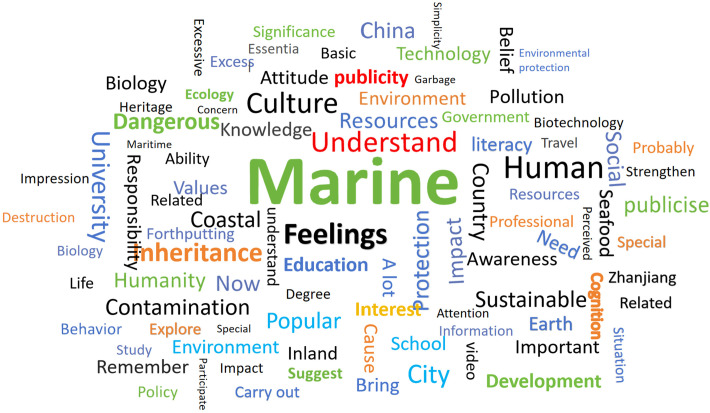
Word cloud of key terms from student interviews. Highlights frequently mentioned words related to the ocean and environmental protection. This figure presents a word cloud based on student interviews, emphasizing common terms such as “ocean,” “environment,” and “protection,” which reflect students’ core concerns.

To investigate participants’ focal concerns regarding marine environmental issues, a word frequency analysis was conducted across the full set of interview transcripts from all 16 participants. The aim was to uncover potential associations among marine environmental beliefs, values, sense of responsibility, and pro-ocean behavioral tendencies. Synonyms were merged during the analysis to consolidate semantically similar terms. The results were visualized in the form of a word cloud (Figure 1), displaying the 50 most frequently occurring terms. Prominent keywords included “ocean,” “feelings,” “environment,” “protection,” and “literacy,” with “ocean protection” and “marine environment” appearing most frequently. These findings indicate strong participant attentiveness to marine-related issues and suggest that emotional connection and cognitive concern for the ocean were dominant themes throughout the interviews.

While the word cloud provided an intuitive visual overview, a deeper understanding of the internal structure of ocean literacy required detailed thematic coding. Therefore, a comprehensive textual analysis was conducted using NVivo to further examine the interrelationships among marine environmental beliefs, values, sense of responsibility, and pro-ocean behaviors. A total of 1,001 unique words were identified, with “ocean” emerging as the most frequent term, accounting for 6.05% of the total word count. This underscores the centrality of ocean-related discourse in participants’ narratives and aligns with the study’s emphasis on enhancing ocean literacy within the framework of environmental education. The high frequency of terms such as “feelings” (3.41%) and “understanding” (1.15%) highlighted the critical role of emotional engagement and cognitive comprehension in motivating pro-environmental behavior. Other notable keywords included “protection” (0.97%), “resources” (0.54%), and “development” (0.56%), reflecting concerns regarding the sustainable use and preservation of marine ecosystems. To support the subsequent theoretical analysis, irrelevant nodes were removed, and the top 20 core terms were identified based on frequency statistics. These are presented in Table 2, providing empirical grounding for thematic interpretation and the construction of the conceptual model.

**Table 2:** Frequency Distribution of Word Occurrence.

No.	Word	Count	Weighted Percentage (%)	Synonyms
1	Ocean	1920	6.05	Ocean
2	Feeling	1089	3.41	Sensation; Emotion; Contact; Feel; Emotion; Taste; Mood
3	Understand	385	1.15	Understand; Comprehend; Recognize; Realize
4	Protect	308	.97	Preserve; Protect; Guarantee
5	Culture	207	.65	Culture
6	Problem	215	.65	Reason Event; Matter; Topic; Problem; Subject; Cause; Self
7	Environment	210	.62	Environment; Situation
8	Influence	200	.60	Achieve; Arrive; Scope; Region; Effect; Extend; Impact; Function
9	Important	179	.56	Important
10	Develop	180	.56	Grow; Develop; Evolve; Develop; Grow
11	Resources	171	.54	Resources
12	Utilize	199	.52	Work; Understand; Utilize; Use; Habit; Apply; Master
13	Place	174	.48	Place; Location; Place; Region; Status; Range; Job; Field; Goal; Area; Position
14	Country	138	.42	Venue; Country; Territory; Foundation; Land
15	Literacy	131	.41	Literacy
16	Feel	138	.39	Hobby; Feel; Experience; Experience; Realization; Experience; Taste
17	Values	123	.39	Values
18	Knowledge	116	.37	Scholarship; Knowledge
19	Humanity	114	.36	Typical; Humanity; Human nature; Someone
20	Like	112	.34	Hobby; Hope; Like; Willing

This table lists the 20 most frequently occurring words in the interview data, along with their weighted percentages and representative synonyms generated through semantic clustering.

### Development of thematic nodes and coding structure

Drawing on semi-structured interviews and established theoretical frameworks related to ocean literacy, this study employed grounded theory methodology to systematically analyze the qualitative data [35]. Through a structured process involving open coding, axial coding, and selective coding, eight core thematic nodes were identified (see Table 3, S1 File–S3). These themes represent key dimensions of university students’ perspectives on the marine environment, encompassing cognitive, emotional, value-based, normative, and behavioral components. Detailed sub-node coding is presented in Table 4. The eight major themes span critical domains such as ocean literacy, human–ocean relationships, environmental values and beliefs, contemporary ecological challenges, marine responsibility, and conservation behavior. In addition, broader thematic areas were explored, including the sociocultural significance of the ocean, marine policy and resource management strategies, and the role of marine education and public awareness. Together, these findings contribute to the construction of a multidimensional and structured analytical framework that reveals the dynamic interrelationships among cognitive, affective, normative, and behavioral dimensions. This framework clarifies how psychological and contextual factors interact to shape individuals’ and groups’ understanding, internalization, and practice of ocean literacy. It highlights the ways in which marine environmental beliefs, emotional engagement, value orientations, and perceived responsibility coalesce to influence pro-ocean attitudes and behaviors, offering theoretical insights for both educational programming and policy design in the realm of marine sustainability.

**Table 3:** Overview of Identified Themes and Their Description.

Theme No.	Theme Name	Description
1	Ocean Literacy	The extent to which individuals understand the ocean’s influence on them and their own influence on the ocean. Encompasses basic ocean science knowledge, systems thinking, and the ability to make informed and responsible decisions regarding the marine environment.
2	Human-Ocean Relations	The perceived connections and interactions between humans and the ocean, including emotional attachment, dependency, and the reciprocal impacts of human activities and marine systems.
3	Marine Environmental Values and Beliefs	Individual and collective value orientations and beliefs regarding marine ecosystems, marine conservation, and the moral significance of protecting ocean health.
4	Marine Environmental Issues	The awareness and understanding of current marine environmental problems, such as pollution, overfishing, habitat destruction, climate change, and biodiversity loss.
5	Marine Responsibility and Behavior	The sense of personal or collective responsibility for marine stewardship, as well as reported or intended pro-ocean behaviors (e.g., waste reduction, beach cleanups, advocacy).
6	Marine Society and Culture	The influence of social structures, traditions, local customs, and cultural heritage related to the ocean, including festivals, myths, and community practices tied to marine environments.
7	Marine Policy and Resource Management	Perceptions and knowledge of laws, regulations, governance mechanisms, and management practices concerning marine conservation and resource utilization at local, national, or international levels.
8	Marine Education and Awareness	The role and effectiveness of formal and informal educational efforts, outreach activities, and public campaigns in raising awareness and fostering ocean literacy in different population groups.

Eight major themes derived from interview analysis, outlining key concepts related to ocean literacy and marine environmental attitudes.

**Table 4:** Sub-node coding situation table.

Sub-nodes	Reference Point/Material Text	Subcategory of Reference Point
Ocean Literacy	216/16	Public knowledge of the ocean (32 nodes)
		Cognition about the ocean (64 nodes)
		Individual ocean literacy levels (52 nodes)
		The role and significance of ocean literacy (24 nodes)
		Components of ocean literacy (44 nodes)
Human-Ocean Relations	226/16	The impact of the ocean on humans (50 nodes)
		Emotions and attitudes towards the ocean (108 nodes)
		Interest and experiences related to the ocean (68 nodes)
Marine Environmental Values and Beliefs	53/16	Composition of marine values and beliefs (24 nodes)
		Impact of marine values and beliefs (29 nodes)
Marine Environmental Issues	105/16	Societal response to marine pollution (47 nodes)
		Public impact of nuclear wastewater incidents (19 nodes)
		Impact of marine environmental issues (39 nodes)
Marine Responsibility and Behavior	180/16	Importance of marine responsibility and conservation behaviors (49 nodes)
		Public attitudes and perceptions towards marine conservation (43 nodes)
		Participation in marine activities (42 nodes)
		Role and significance of marine behaviors (23 nodes)
		Challenges and strategies in marine conservation actions (23 nodes)
Marine Society and Culture	73/16	Importance of marine society and culture (28 nodes)
		Protection and inheritance of marine cultural heritage (45 nodes)
Marine Policy and Resource Management	150/16	Marine policy and international cooperation (43 nodes)
		Public perceptions of marine resource utilization (36 nodes)
		Marine resource development and conservation (71 nodes)
Marine Education and Awareness	148/16	Current state of marine education (27 nodes)
		Approaches to marine popular science education (43 nodes)
		Public awareness of the ocean (49 nodes)
		The role and significance of marine education (29 nodes)

Subcategories and reference frequencies for each theme, based on qualitative coding across 16 participants.

### Interview research findings

#### (I) Multidimensional perspectives on ocean literacy.

Analysis of the 44 coded nodes revealed that participants generally conceptualized ocean literacy as a multidimensional construct. In addition to scientific knowledge, students emphasized four additional dimensions: environmental ethics, conservation behavior, policy understanding, and critical thinking. All 16 interviewees referenced at least three or more core components of ocean literacy, suggesting a comprehensive and behaviorally oriented understanding of the concept. Most participants perceived ocean literacy not only as the mastery of ocean-related knowledge but also as an expression of environmental awareness, sustainable resource use, and active engagement in marine conservation. Several participants specifically underscored the importance of respecting marine ecosystems and promoting sustainable development, framing ocean literacy as an internal transformation process—from environmental attitudes to concrete actions. Regarding the breadth and depth of public understanding of ocean science, 12 out of 16 participants were able to recall basic knowledge such as marine ecosystems, biodiversity, and ocean currents. However, only five interviewees mentioned more complex, systems-level topics such as ocean acidification or the marine carbon cycle. These findings suggest that while most students possess foundational knowledge of ocean science, their understanding of systemic ecological processes remains limited.

Cognitive and Emotional Connection with the Ocean: Participants demonstrated a broad awareness of the ocean’s physical and biological characteristics, as well as its influence on global climate and ecological systems. Nevertheless, comprehension of more abstract or complex environmental issues was generally insufficient. All 16 participants explicitly discussed individual differences in ocean literacy, contributing to 52 coding references. These differences were evident in both cognitive understanding and behavioral expression. Students with higher levels of ocean literacy displayed stronger scientific comprehension and were more actively involved in conservation practices. By contrast, those with lower levels of literacy tended to focus on surface-level information and reported fewer pro-environmental behaviors. Perceived Role and Significance of Ocean Literacy: Thirteen participants referred to the functional role and societal importance of ocean literacy, yielding 24 distinct coding references. The majority of these emphasized its environmental significance, particularly its potential to foster a sense of environmental responsibility and to motivate concrete conservation behaviors. Participants widely viewed ocean literacy as a critical driver of both individual and collective action toward marine sustainability.

*A2: “I think it’s quite important, and as a prerequisite for ocean literacy, it’s essential to have a correct understanding. That is, recognizing that the ocean itself is fragile; it is not as strong as it appears.”*

*A11: “The ocean knowledge I’ve encountered is quite limited, and I feel there is also a lack in the spread of this knowledge online. The efforts to popularize it are not sufficient.”*

*A7: “I believe the ocean is the origin of life, and everything on our planet is nurtured from the ocean. So, I see the ocean as the cradle of life for all of humanity and also as a mysterious presence.”*

#### (II) Human-ocean relationships.

Perceptions of the Ocean’s Influence on Humanity: Analysis of 50 coded references revealed that 13 participants explicitly discussed the ocean’s comprehensive and multidimensional influence on human beings. The ocean was commonly perceived not only as the origin of life but also as a symbolic frontier for human exploration, representing a longing for the “unknown” and a desire for “freedom.” These expressions underscore the ocean’s role in shaping ecological consciousness, self-awareness, and environmental responsibility. Participants’ reflections indicated that the ocean is understood not merely as a physical element of the natural world, but also as an internalized dimension of human cognition, personal identity, and cultural imagination. This cognitive framing aligns closely with key components of ocean literacy—particularly environmental awareness and a sense of personal responsibility.

Emotional and Attitudinal Dimensions Toward the Ocean: A total of 108 coded references from all 16 participants addressed emotional and attitudinal responses toward the ocean. The range of emotions expressed included awe, affection, fear, and concern. Some participants described the ocean as a source of psychological comfort and healing, while others expressed anxiety regarding its unpredictability and the threats posed by marine pollution. These nuanced emotional responses illustrate the affective dimension of ocean literacy and highlight how feelings of reverence, attachment, or vulnerability toward the ocean may shape environmental attitudes and behavioral intentions. Ocean-Related Interests and Experiential Engagement: All 16 participants shared personal experiences and interests related to the ocean, generating 78 coded references. Commonly mentioned activities included swimming, diving, sailing, and coastal exploration. These were described not only as recreational pursuits, but also as meaningful opportunities to connect with nature and deepen one’s emotional bond with the marine environment. Such experiential engagement reflects the behavioral intention component of ocean literacy—where emotional identification with the ocean fosters a greater willingness to participate in marine-related initiatives and engage in pro-environmental behaviors.

*A3: “The ocean is closely linked with our planet. If the ocean is polluted, our Earth’s water resources will also be polluted, which would affect the ecological cycle. This could then have a significant impact on our planet.”*

*A1: “Being enveloped in nature, I feel unified with the water and the wind, and it’s very relaxing. My feelings towards the ocean are definitely fond; I particularly like the ocean.”*

#### (III) Ocean policy and resource management.

Perspectives on Ocean Policy and Resource Management: A total of 150 coded references were identified in discussions concerning ocean policy and resource management, indicating that this topic was of significant concern across all 16 participants. Specifically, 14 interviewees emphasized the importance of international cooperation in the sustainable governance of marine resources, contributing 43 coding references. Many participants expressed the view that global challenges such as marine pollution and overfishing necessitate transboundary co-management mechanisms and the establishment of marine protected areas (MPA). While international frameworks—such as the United Nations 2030 Agenda for Sustainable Development—provide strategic direction for global action [1,36], several participants observed that China continues to face challenges in areas such as marine legislation and law enforcement, underscoring the need for stronger implementation and broader public engagement.

Regarding public attitudes toward marine resource utilization, all 16 participants expressed diverse perspectives, accounting for 36 coded references. On one hand, they acknowledged the economic value of marine resources; on the other, they voiced concerns about the irreversible ecological damage resulting from overexploitation. The participants’ suggestion of an “annual fishing ban” reflects a perceived need for enhanced Province-level regulatory intervention, aligning with the theme of policy-driven conservation expectations. Most participants regarded education and public awareness as critical strategies for achieving a balance between resource development and environmental conservation. When discussing the tension between marine resource exploitation and ecological protection, all participants contributed to 71 coded references. Many stressed that policy guidance and technological innovation must function synergistically. Some interviewees also cited European experiences, suggesting that the effective institutionalization of marine resource governance requires an integrated approach combining policymaking, public education, and regional cooperation. Overall, participants demonstrated a nuanced and multilayered understanding of ocean policy and resource management, encompassing global frameworks and national responsibilities as well as local practices. Their reflections collectively highlight three key dimensions of ocean literacy: policy awareness, resource stewardship, and a sense of environmental responsibility.

*A9: “The government and international organizations should take responsibility. Public awareness is often influenced by international and governmental actions. If national governments promote marine conservation, people will fully understand the importance of protecting the oceans, and they will act accordingly. The effort to promote this is quite effective.”*

*A15: “The utilization of marine resources should be planned sensibly, and excessive development should be avoided. Intense exploitation can damage the ecosystem, leading to irreversible consequences.”*

*A7: “Sustainable development is crucial. Each year, there should be a fishing ban period to allow marine life to reproduce more abundantly during this time. This can help restore populations to their previous states and allow us to continue harvesting resources. We should adhere to the principles of sustainable development and protect our marine resources.”*

#### (IV) Ocean values and beliefs.

A total of 53 coded references were identified in discussions related to participants’ marine environmental values and beliefs. This included 24 references from 13 participants describing the structure of these values, and 29 references from all 16 participants addressing their influence on behavior. Most interviewees generally agreed that the ocean is an irreplaceable natural resource essential to planetary health, emphasizing the importance of biodiversity conservation for maintaining global ecological stability. Many participants expressed biospheric value orientations and ecocentric perspectives, affirming that humanity has a moral obligation to protect the marine environment beyond considerations of self-interest. The interviews further revealed that ocean conservation education plays a significant role in shaping environmental values and facilitating behavioral transformation. Participants who had received systematic marine education were more likely to engage in pro-environmental actions such as supporting no-fishing zone policies, participating in beach cleanups, and reducing the use of single-use plastics. This educational exposure not only enhanced cognitive understanding but also reinforced participants’ sense of environmental responsibility and willingness to act.

Social networks also emerged as important contributors to the formation of environmental values. Several participants noted that their pro-environmental behaviors were influenced by family members, peers, and student organizations, underscoring the role of group norms in encouraging individual engagement in marine conservation. Notably, geographic context appeared to influence value orientations: participants from coastal regions, owing to their proximity to and frequent interaction with the ocean, demonstrated stronger intentions to protect it. In contrast, inland students exhibited lower levels of marine awareness and weaker behavioral intentions. Taken together, these findings suggest that the internalization of ocean values and beliefs reflects not only changes in knowledge and attitudes but also the combined influence of educational interventions and social context. The results indicate that enhancing context-sensitive ocean education, fostering collective pro-environmental norms, and strengthening public awareness of environmental responsibility are effective strategies for promoting sustainable ocean-related behaviors.

*A11: “For marine conservation, it is the belief in the value of the ocean that can restrain oneself from harming it. You must respect, honor, and revere the ocean, and then protect it. Those who have been in contact with the ocean or understand it possess a firmer set of values and beliefs about protecting it.”*

*A13: “Beliefs and values are certainly important; they affect your worldview and, consequently, the actions you take in everyday life. These beliefs and values, when presented internationally, involve actions related to the protection and development of the oceans, ultimately aiming to conserve them.”*

#### (V) Marine responsibility and behavior.

A total of 180 coded references were identified under the theme of marine responsibility and behavior, encompassing five interrelated subthemes. First, 15 participants (49 references) emphasized the importance of marine responsibility and pro-environmental behavior. They highlighted that individuals could fulfill their responsibility for ocean conservation through everyday actions such as reducing single-use plastics and participating in beach cleanups. Most participants believed that enhancing both personal and collective responsibility is essential for fostering environmentally sustainable behaviors. Second, all 16 participants (43 references) discussed public attitudes and emotional responses toward marine conservation. While they generally expressed positive sentiments—such as concern, appreciation, and hope—many also noted existing gaps in public understanding and in the capacity to translate awareness into concrete action. Participants emphasized that increasing public engagement and offering immersive, hands-on experiences are critical for facilitating meaningful behavioral change. Third, all 16 participants (42 references) addressed the frequency and nature of participation in marine-related activities. Participation levels were reported to vary according to geographic location, educational background, and individual interest. Students from coastal regions exhibited higher levels of exposure to and involvement in ocean-related activities compared to their inland counterparts. Fourth, 10 participants (23 references) reflected on the broader significance of pro-ocean behavior. They observed that such actions not only contribute to ecological restoration but also support economic development and enhance human well-being, underscoring the multifaceted societal value of marine environmental engagement. Finally, 12 participants (23 references) identified key challenges and proposed strategies for improving marine conservation efforts. Reported challenges included insufficient funding, inadequate enforcement of environmental regulations, and low levels of public participation. Recommended solutions involved strengthening environmental education and outreach, improving policy frameworks, and fostering multi-stakeholder collaboration.

Overall, participants widely acknowledged the importance of marine responsibility and expressed a general willingness to engage in ocean-protective actions. However, significant variation was observed in the depth of knowledge and breadth of participation. These findings underscore the urgent need to strengthen ocean literacy, promote participatory engagement, and enhance institutional support mechanisms to advance sustainable marine behaviors.

*A2: “If we don’t have a sense of responsibility to protect the ocean, it will get polluted. Even a small action can have a big impact on the ocean.”*

*A8: “The general public’s concern for the ocean is not very high, and some people are not even aware of it, with a lot of pollution still being dumped into the sea. A sense of responsibility is very important, and more people should be encouraged to feel responsible for the ocean; the effort of a few is always not enough.”*

*A3: “I’ve participated in a beach clean-up organized by my school, which I found very meaningful. Sometimes I like walking on the beach, and a couple of days ago, I also went to a shipyard with an activity and went aboard a ship.”*

*A14: “A sense of responsibility towards the marine environment is crucial for ocean protection but faces challenges and issues in practice. It is advisable to start small, focusing on everyone’s voluntary actions. Promoting environmental protection extensively, perhaps by setting up eco-friendly slogans in entertainment venues, could raise everyone’s environmental awareness.”*

#### (VI) Marine society and culture.

A total of 73 coded references were identified under the theme of marine society and culture, encompassing two primary dimensions. First, all 16 participants (28 references) emphasized the importance of the ocean in shaping social and cultural structures. Participants widely acknowledged that the ocean profoundly influences the lifestyles, languages, festivals, and educational content of coastal communities, while also indirectly shaping cultural perceptions in inland regions. As a conduit for transportation and trade—particularly through historical routes such as the Maritime Silk Road—the ocean has facilitated intercultural and economic exchanges between China and other nations, contributing to the development of a distinctive maritime cultural landscape. Second, 14 participants (45 references) expressed concern regarding the preservation and transmission of marine cultural heritage. They underscored the value of intangible cultural assets such as traditional fishing village practices, maritime history, and the beliefs surrounding Mazu, the Chinese sea goddess. Participants noted that many aspects of this traditional knowledge and practice are increasingly at risk of disappearing amid rapid modernization and urban development. To address these challenges, they called for stronger cultural preservation policies and more robust educational mechanisms to ensure the continuity of marine heritage and to protect the cultural identity associated with it.

*A3: “It’s quite important in cultural aspects, with many literary works and paintings inspired by the ocean. So, it’s quite significant in the arts.”*

*A5: “It’s definitely worth protecting! It’s also a part of Chinese traditional culture. Some coastal communities have depended on fishing for generations, and these cultural traditions passed down are a valuable heritage.”*

*A6: “I’ve heard about Mazu, and many people believe in her, especially in Fujian. The Maritime Silk Road is itself a cultural heritage that facilitated the exchange between Eastern and Western civilizations, not only allowing for trade but also for the exchange of technologies.”*

#### (VII) Marine education and awareness.

A total of 148 coded references were identified under the theme of marine education and awareness, encompassing four key areas: the current status of marine education, pathways for public science communication, levels of public awareness, and the broader educational function of ocean literacy.

First, 14 participants (27 references) highlighted significant regional and disciplinary imbalances within China’s current marine education system. In particular, non-coastal regions were reported to have limited access to marine-related content. Many existing courses were described as overly theoretical, lacking practical engagement, interactivity, and localized relevance, which in turn hindered students’ interest in marine science and limited the depth of their understanding.

Second, regarding pathways for marine knowledge acquisition, all 16 participants (43 references) identified a variety of channels through which the public gains access to ocean-related information. These included formal school curricula, short-video platforms, official WeChat accounts, television documentaries, and science exhibitions. However, many participants also criticized the fragmented and entertainment-oriented nature of current marine science communication. Some content was perceived as lacking scientific rigor and coherence, potentially contributing to public misunderstanding. Hands-on, context-rich experiences—such as visiting aquariums or participating in coastal ecological restoration projects—were seen as effective approaches for bridging knowledge gaps and fostering meaningful engagement.

Third, in terms of public awareness, participants generally believed that societal concern for marine conservation remains insufficient. Residents of inland regions, in particular, were noted to have lower awareness of marine issues and limited understanding of the ecological functions of ocean systems and their significance for human well-being (49 references).

Finally, 13 participants (29 references) emphasized that marine education plays a pivotal role not only in enhancing individual environmental awareness and responsibility, but also in fostering pro-environmental behavioral intentions. At the collective level, marine education was seen as a catalyst for building social consensus around environmental policies and advancing sustainable ocean development. Overall, participants strongly affirmed the integrative function of marine education in promoting public ocean literacy, shifting environmental perceptions, and guiding behavioral action. They advocated for comprehensive reforms to educational systems and the enhancement of science communication mechanisms to cultivate a scientifically informed and sustainability-oriented public understanding of the ocean.

*A2: “In my primary, middle, and high school, they only said not to play by the riverbank, not the seaside, and there wasn’t much spread about this. There was also no talk of marine education.”*

*A16: “As a child, I learned about the ocean through books, like ‘A Hundred Thousand Whys’ and other marine science popular books. Later on, I gradually learned more through the internet and science videos, which also delved deeper.”*

*A9: “I don’t think it’s very good, many people mainly see the ocean as just beautiful, right? In reality, there isn’t a well-formed, systematic awareness about the ocean. I think the level of ocean awareness among the Chinese public is not sufficient; it’s quite weak.”*

*A6: “There’s a lot of trash, especially on the beaches where many people go to play. You see trash floating around by the seaside, which shows that their ocean awareness is quite weak.”*

#### (VIII) Marine environmental issues.

Under the theme of marine environmental issues, 15 participants discussed public responses to ocean pollution (47 references), 12 participants addressed the social impact of Japan’s nuclear wastewater discharge incident (19 references), and 15 participants reflected on the broader consequences of marine environmental degradation (39 references). Most interviewees expressed deep concern regarding ocean pollution, reporting that such incidents significantly reduced their willingness to engage in marine-related activities—particularly in contexts where the ocean was perceived as “unclean” or “unsafe.” Participants noted that public reactions to pollution extended beyond ecological considerations to encompass health and economic concerns. Several respondents voiced apprehension about the potential impacts on seafood safety and coastal tourism, suggesting growing public awareness of the multifaceted threats posed by marine pollution to ecosystems, economic stability, and public health.

With respect to Japan’s nuclear wastewater discharge event, 12 participants observed that the incident significantly heightened societal attention to marine environmental protection, serving as a powerful catalyst for environmental awareness. However, the interviews also revealed that such concern tends to be event-driven and short-lived. As media attention declines, the momentum behind individual pro-environmental behaviors often dissipates. This finding highlights the challenge of transforming emotionally charged responses to environmental crises into sustained behavioral change. In terms of broader marine environmental degradation, 15 participants emphasized the far-reaching consequences of pollution for marine ecosystems, biodiversity, fisheries, tourism, and food security. Several noted the risk of harmful substances—such as heavy metals—accumulating in seafood, raising public health concerns and contributing to heightened social anxiety. While participants generally demonstrated strong sensitivity to marine environmental issues; however, most admitted that their own conservation behaviors remained basic, often limited to actions such as refraining from littering. A more systematic sense of environmental responsibility and behavioral commitment was largely absent. These findings point to a clear “attitude–behavior gap” in public engagement with ocean protection. Although environmental awareness is relatively high, meaningful and sustained action remains limited. This underscores the urgent need for targeted educational interventions and policy initiatives to bridge this gap and cultivate deeper, long-term public engagement in marine environmental stewardship.

*A11: “I think marine protection is very necessary. Like the nuclear wastewater, it has now flowed into our waters from Japan. Last year, when Japan discharged nuclear pollution, we asked our teachers whether it would affect humans because we work at sea and spend a lot of time there, wondering if it could impact us?”*

*A5: “If the ocean is polluted, our Earth’s water resources will also be polluted, and then it would affect the ecological cycle, which could have a significant impact on our planet. I think humanity should not overly explore the ocean, avoid damaging the ecosystem, and ensure that resource extraction does not destroy the ecology.”*

## Discussion

This study, based on semi-structured interviews, systematically revealed the multidimensional structure of ocean literacy among Chinese university students. Five core components were identified: scientific knowledge, environmental ethics, behavioral intentions for conservation, policy understanding, and critical thinking. These findings provide additional empirical support for the conceptualization of ocean literacy as a multidimensional construct [3,37], and align with recent interdisciplinary reviews that emphasize the expanding role of ocean literacy in marine education [20,38]. With respect to the current state of knowledge and its implications for education, most participants demonstrated a solid grasp of basic oceanographic concepts. However, their understanding of more complex systemic processes—such as the carbon cycle and ocean acidification—was limited. This cognitive gap mirrors findings from other studies conducted in Asian contexts [29,39], and reinforces conclusions from broader reviews of ocean education in the Asia-Pacific region, which emphasize the urgent need for interdisciplinary curricula and the cultivation of systems thinking skills [40].

The findings also underscore the pivotal role of emotional connection and experiential engagement in shaping and enhancing ocean literacy. Many participants described the ocean as a source of psychological comfort and belonging—resonating with prior research by Brennan and Tam [3,41] and further supported by emerging evidence from neuroscience and environmental psychology, which highlights the influence of affective experiences on the transition from environmental awareness to action [42,43]. Notably, students from coastal areas—due to their frequent direct contact with the ocean—were more likely to translate emotional attachment into pro-environmental behaviors. The integration of aesthetic experiences, cultural identity, and ecological value perception appeared to foster deeper conservation commitment and promote psychological well-being [44,45]. These findings are consistent with evolutionary psychology perspectives on human affinity for aquatic environments and the well-established mental health and environmental benefits of “blue space” exposure [17,46]. Accordingly, future marine education efforts should place greater emphasis on integrating emotional and experiential components with cognitive instruction. The development of interactive, immersive, and place-based learning opportunities may be essential for enhancing public awareness and fostering long-term engagement in marine conservation.

Furthermore, marine environmental values and beliefs emerged as critical determinants of pro-environmental behavior among Chinese university students. Consistent with the Value-Belief-Norm (VBN) theory, individuals who possess strong marine conservation values are more likely to develop heightened environmental awareness and a sense of moral obligation, which in turn promotes active engagement in ocean-protective behaviors [13]. These finding echoes prior research indicating that ecological responsibility, particularly in higher education settings, can be significantly strengthened through systematic marine education and experiential learning opportunities [47]. Students from coastal regions generally exhibited a stronger sense of environmental responsibility, consistent with prior research suggesting that geographic proximity to marine environments significantly influences environmental cognition and behavior [2,48]. In the domain of marine policy and resource management, interviewees frequently emphasized the importance of cross-regional and international collaboration in addressing critical issues such as marine pollution and resource overexploitation—an insight supported by global research on ocean governance [1]. While some participants demonstrated awareness of existing marine protection policies, skepticism regarding policy implementation and obstacles to public participation remained prominent. These concerns echo recent comparative policy analyses, which identify public engagement and policy transparency as essential for improving environmental governance outcomes [49].

Cultural practices, including local beliefs, community festivals, and maritime heritage traditions—were also perceived as effective in fostering a stronger sense of marine responsibility [50]. Additionally, the role of social learning environments, such as family, peer networks, and digital platforms, was frequently highlighted. Recent findings by Xie et al. demonstrate that digital social networks and collective norms significantly influence the environmental behaviors of Chinese youth, reinforcing the conclusions of this study [51]. The protection and transmission of marine cultural heritage were also viewed as critical pathways for strengthening public environmental identity and promoting inclusive marine governance [52]. Interactive learning environments—such as marine museums and aquariums—have been empirically shown to effectively enhance public ocean literacy [8]. Many participants expressed a strong interest in more experiential and participatory learning formats, supporting the view that educational innovation plays a vital role in fostering ocean awareness [19]. Despite this enthusiasm, several participants reported a disconnect between theoretical content and practical engagement, as well as a lack of interaction in current educational delivery. They suggested that emerging technologies such as virtual reality (VR) could improve learning motivation and foster systems thinking. Recent empirical studies have demonstrated the effectiveness of immersive and blended marine education approaches in enhancing both knowledge acquisition and pro-environmental behavior [53,54]. Taken together, these findings suggest that achieving sustainable behavioral change among university students requires the integration of cognitive instruction, emotional engagement, and practical participation. Such a holistic approach to marine education is essential for translating awareness into long-term conservation behavior.

This study found that sudden environmental events—such as the release of nuclear wastewater—can temporarily raise public environmental awareness but often fail to produce sustained behavioral change. This finding supports longstanding assertions in environmental psychology that systematic education and consistent policy interventions are essential for achieving long-term impact [14,55]. In contrast, participants expressed greater trust in educational interventions and routine pro-environmental practices—such as participating in beach cleanups and reducing single-use plastic consumption—as effective strategies for enhancing both individual and collective responsibility and fostering sustained engagement in conservation behavior [48]. Additionally, participants viewed marine protected areas, resource co-management, and international cooperation as critical tools for addressing global marine challenges—an assessment that aligns with successful case studies in Europe and other regions [1]. In the Chinese context, participants emphasized the importance of aligning national strategies with local cultural and social practices, strengthening policy enforcement, enhancing public participation, and implementing Goal 14 of the United Nations 2030 Agenda for Sustainable Development [6].

While this study offers a comprehensive examination of the psychological mechanisms linking ocean literacy and pro-environmental behavior among Chinese university students, several limitations must be acknowledged. First, the sample was drawn primarily from selected coastal and inland universities in China, which may limit the representativeness and generalizability of the findings to the broader student population. Second, data collection relied on semi-structured interviews, which may be subject to social desirability bias and participants’ subjective interpretations—potentially limiting insight into the complexity and fluidity of behavioral change processes. Third, the cross-sectional nature of the study precludes the ability to assess the long-term effects of educational interventions or emotional experiences on behavioral transformation. To address these limitations, future research could be improved in the following ways:

(1) Broaden the sample scope to include university students from more diverse geographic regions, academic disciplines, and cultural backgrounds to enhance the generalizability and external validity of the findings.
(2) Adopt mixed-method research designs—such as combining quantitative surveys with qualitative interviews or implementing longitudinal tracking studies—to strengthen methodological rigor, improve objectivity, and enhance theoretical robustness [56].
(3) Incorporate longitudinal approaches to systematically evaluate the long-term effects of educational innovations, policy reforms, and community-based interventions on university students’ pro-environmental behaviors.
(4) Further investigate mediating and moderating mechanisms, such as emotional connectedness to the ocean, the influence of digital technologies, and other emerging psychosocial factors that may shape the transformation of ocean literacy into concrete conservation actions.

## Conclusion

Through qualitative analysis, this study systematically revealed the critical role of ocean literacy in promoting pro-environmental behavior among Chinese university students. The findings demonstrate that five core components—scientific knowledge, environmental ethics, behavioral intention, policy understanding, and critical thinking—collectively support students’ comprehensive understanding of and active engagement with marine environmental issues. However, students’ current depth and systematic understanding of ocean science among students remains limited, thereby constraining the long-term sustainability of their conservation efforts. This underscores the imperative to strengthen marine education systems grounded in scientific literacy and critical thinking.

Emotional connectedness and direct experiential learning were identified as powerful drivers of environmental awareness and behavioral transformation. Effective marine policies, responsible resource management, and international cooperation were also identified as indispensable for addressing global marine environmental challenges. In addition, the preservation and transmission of marine cultural heritage were recognized as having unique value in cultivating individual responsibility and fostering collective environmental action.

The study highlights the need to integrate cultural relevance, social participation, and interactive experiential learning into future ocean education practices to enhance both public ocean literacy and behavioral capacity. It is recommended that future marine education initiatives should adopt more practical and innovative pedagogical approaches—such as virtual reality and immersive learning environments—to improve student engagement, comprehension, and long-term behavioral commitment.These findings provide both theoretical insights and practical pathways for optimizing ocean education, strengthening public engagement, and deepening international cooperation—ultimately contributing to the realization of sustainable ocean governance goals.

## Supporting information

S1_File Anonymized Transcript Excerpts with Theme Mapping. (PDF)

S2_File Sample Interview Transcript (Anonymized). (PDF)

S3_File Thematic Codebook and Representative Quotes. (PDF)
