# Supplementary material for: The promotive effect of ocean literacy on marine conservation behavior: A qualitative study based on Chinese university students
Source: PLoS One. 2025 Aug 8;20(8):e0323510. doi: 10.1371/journal.pone.0323510 (PMC12333999; doi:10.1371/journal.pone.0323510)
Supplement: S1_File — (PDF) [file pone.0323510.s001.pdf]

S1_File. Anonymized Transcript Excerpts with Theme Mapping

Representative Quotes Categorized by Thematic Code

Source: Semi-structured Interview, Participant A1、A9、A14.

Note: All personally identifiable information has been removed. Quotes are translated from Chinese into English and thematically labeled.

A1:

Theme	Quote	Original Quote (Chinese)
T1. Ocean Knowledge and Understanding	“The ocean is very vast and powerful. It can generate waves and typhoons, and it's where seafood comes from.”	“海洋它是一个很大很强的力量，会刮风，会有台风，会有浪，还有我们吃的海鲜。”
T2. Ocean Activities and Personal Experience	“I participated in the sailing team activities at our university, and we also joined a national sailing competition.”	“我们小校有广东海洋大学帆船队……我还是比帆赛了……还有去厦门参加全国大学生帆船联赛。”
T3. Emotional Attachment to the Ocean	“I feel it's like nature itself... I really enjoy being close to it. Watching the waves gives me peace.”	“我感觉他就是一种自然吧……就很喜欢跟他的一种相处，看海浪就会很平静。”
T4. Perception of Ocean Pollution	“There is still some trash on the beach... plastic and bottles. I think we should raise awareness about marine pollution.”	“我觉得那个海滩上还是有垃圾的……就是塑料、瓶子……我觉得还是要提高一下大家的环保意识。”
T5. Policy and Regulation Awareness	“I think there should be an annual fishing ban period... to let the ocean rest and recover.”	“我觉得每年应该有一个禁渔期……让海洋休息一下。”
T6. Media and Information Sources	“I saw some documentaries on marine conservation online... and also some videos about plastic harming marine animals.”	“我在网上看到一些关于保护海洋的纪录片……还有一些关于塑料对海洋动物伤害的视频。”
T7. Ocean Education and School Curriculum	“We had a marine science elective once, but I feel it was too general and lacked real-life examples.”	“我们上过一次海洋科学的选修课，但我觉得太泛泛了，没有什么实际的例子。”
T8. Marine Environmental Responsibility	“I think everyone should do their part to protect the ocean, even if it's just picking up trash when you go to the beach.”	“我觉得每个人都应该为保护海洋出一份力……哪怕就是去海边的时候捡一下垃圾。”

A9:

Theme	Quote (English)	Quote (Chinese)
T1. Ocean Knowledge and Understanding	I think the ocean is vast and powerful... It creates waves, typhoons, and provides seafood. We even learned about combustible ice and offshore oil in middle school.	海洋它是一个很大很强的力量，会刮风，会有台风，会有浪，还有我们吃的海鲜。我们初中就知道有个可燃冰，还有石油。
T2. Emotional Connection to the Ocean	Seeing the open ocean for the first time in Zhanjiang was amazing. It felt endless and beautiful—really shocking compared to inland lakes.	我第一次看到海是在湛江的角尾，感觉跟内海很不一样，就是没有边的，特别震撼！
T3. Ocean Information Sources	I usually get ocean-related info from WeChat public accounts like the South Sea Fleet, and from documentaries or news videos I saw as a child.	我通常通过南海舰队这样的公众号，还有小时候看的纪录片和新闻视频了解海洋的信息。
T4. Interest in Marine Life	I'm especially interested in marine life—like corals, algae, and deep-sea creatures. They're mysterious and beautiful, even if sometimes scary.	我特别对海洋生物感兴趣，比如珊瑚、藻类、深海生物，觉得它们神秘又好看，虽然有时候有点吓人。
T5. Personal Marine Experience	Taking a boat to Laozhou Island left a deep impression. Floating on the sea felt different from being on land or in a plane.	坐船去老洲岛的经历让我印象深刻，海上漂浮的感觉跟在陆地或飞机上都不一样。
T6. Perceived Ocean Literacy Level	I think I only have average knowledge about the ocean. I learn things passively when they appear in class or in my social feeds.	我觉得自己对海洋的了解一般，只有在课堂上或者刷公众号碰巧看到时才会学点东西。
T7. Views on Ocean Education	Marine education isn't widespread enough. Most people only associate the ocean with beaches and sunshine, not with typhoons or climate.	海洋教育普及度不够，大多数人只想到阳光和沙滩，没意识到台风或气候这些更深层次的问题。
T8. Marine Environmental Responsibility	I've picked up trash on the beach before. Closer to human activities, the ocean tends to be more polluted. Everyone's small actions matter.	我之前在海边捡过垃圾，越靠近人类活动区污染越严重，每个人的小行为都会产生影响。
T9. Public Concern and Problems	People focus on big problems like Japan's wastewater, but ignore their own behavior. There's a 'not my problem' mindset.	人们对日本核废水这种大问题很关注，但对自己行为却很忽视，觉得事不关己。
T10. Views on	Policy enforcement sometimes feels	有时候感觉政策执行只是做做样子，

Policy Implementation	performative. I saw inspectors taking photos, but sewage treatment was clearly insufficient.	我看到检查人员拍照，但实际的污水处理很不到位。
T11. Sustainable Marine Development	We should replace nonrenewable resources gradually and avoid overfishing. Ecosystem protection must come with resource use.	我们应该逐步替代不可再生资源，不能过度捕捞，资源开发必须和生态保护并重。
T12. Marine Values and Beliefs	The ocean connects all countries. Marine protection is everyone's responsibility because issues like pollution spread globally.	海洋把所有国家都连接在一起，污染等问题会全球扩散，所以每个人都有责任去保护海洋。

A14:

Theme	Quote (English)	Quote (Chinese)
T1. Ocean Knowledge and Recognition	The ocean is part of nature, and we need to live in harmony with it. I've lived close to the sea all my life.	海洋是大自然的一部分，我们要与自然和谐共处。我从小就住在靠海的地方。
T2. Environmental Observation and Pollution Awareness	The environment near Yantian Port is poor. You can see floating plastic, oil, and dirty things from ships.	盐田港那边环境比较差，可以看到塑料、油渍和从船上掉下来的脏东西。
T3. Interest in Marine Issues	I'm very interested in marine issues, especially after the nuclear wastewater news from Japan.	我对海洋问题很感兴趣，特别是日本核污染排放的新闻之后。
T4. Marine Information Sources	I learn about the ocean from observation and public accounts, but rarely from books.	我主要通过观察和公众号了解海洋内容，很少看书。
T5. Personal Experience and Attitude	I maintain awe toward the ocean. It's deep and mysterious, and crucial for national development.	我对海洋保持敬畏，它深不可测，是国家发展的关键。
T6. Knowledge Level and Literacy	My ocean knowledge is average. I have some understanding, but not in depth.	我的海洋知识水平中等，懂一点但不多。
T7. Views on Public Awareness	The public has limited understanding of the ocean, and there's not enough promotion.	公众对海洋的了解有限，宣传也不够。
T8. Marine Education	Marine education is necessary so everyone can understand the seriousness of ocean issues.	很有必要开展海洋教育，让大家了解问题的严重性。
T9. Marine	With a sense of responsibility, people will	有责任感的人会像对待自己家

Environmental Responsibility	treat the sea like their own home and keep it clean.	一样保持海洋清洁。
T10. Marine Protection Behavior	Most people don't throw garbage into the sea on purpose. Pollution often comes from accidents or carelessness.	大多数人不会故意往海里丢垃圾，很多污染是意外或疏忽造成的。
T11. Policy and Governance	Policies like fishing bans are good. They help protect marine species and ecosystems.	像禁渔期这样的政策很好，能保护海洋生物和生态系统。
T12. Values and Beliefs	Belief leads to action. If we believe in protecting the ocean, we'll act on it.	信念决定行动，如果我们有保护海洋的信念，就会采取行动。
