# Supplementary material for: The promotive effect of ocean literacy on marine conservation behavior: A qualitative study based on Chinese university students
Source: PLoS One. 2025 Aug 8;20(8):e0323510. doi: 10.1371/journal.pone.0323510 (PMC12333999; doi:10.1371/journal.pone.0323510)
Supplement: S2_File — (PDF) [file pone.0323510.s002.pdf]

S2_File. Sample Interview Transcript (Anonymized)

Note: This file contains anonymized excerpts from three semi-structured interviews (Participants A1, A9, A14). All identifying details have been removed in accordance with research ethics standards. Each excerpt includes basic demographic context and highlights representative statements on themes related to ocean literacy, environmental concern, and marine values.

Participant A1

Gender: Female
Academic Year: Year 2

Excerpt:

The first time I saw the ocean was in Zhanjiang, and it felt completely different from the inland sea. It was vast and endless—it really shocked me. I had always dreamed of seeing the ocean since I was a child, and that moment felt like a dream come true. Now that I study in a coastal university, I get to interact with the sea more often. I even joined the sailing team and participated in national competitions. These experiences deepened my emotional connection to the ocean.

I′ve also noticed pollution on the beaches—trash like bottles and plastic bags are still common. I think this shows that while we are close to the ocean, our sense of environmental responsibility still needs to improve. Marine knowledge is often acquired passively, such as when I see news articles, videos, or course content. I think marine education should be more immersive and begin earlier, even in elementary or secondary schools.

When talking about marine values, I believe we should maintain a sense of awe and respect. The ocean is powerful but fragile. It can bring typhoons and destruction, but it also sustains life. Protecting the ocean isn′t just about laws—it′s about attitude, belief, and daily action.

Sometimes I feel conflicted when I see the beach being used for commercial tourism. On the one hand, it promotes economic development, but on the other, it causes serious environmental degradation. There should be more environmentally-friendly ways to promote marine tourism.

Participant A9

Gender: Male
Academic Year: Year 3

Excerpt:

Living close to the sea all my life, I′ve developed a natural connection to it. I think of the ocean as something vast and mysterious—it provides oxygen, regulates climate, and supports countless species. But its health is clearly declining. I often see trash along the shoreline and smell oil when walking by

the docks. It's frustrating, because I don't think most people intentionally litter—it's more about a lack of awareness or infrastructure.

I believe ocean education should be strengthened, not just for students in related majors like mine, but for everyone. When people truly understand the ocean's value, they're more likely to protect it. Many people I know—even from non-coastal areas—became more aware after events like the Japan wastewater controversy. Social media plays a role in raising awareness, but it's inconsistent.

In terms of ocean literacy, I'd say I have a moderate level of knowledge. I'm familiar with ocean trade routes, basic marine ecology, and the concept of sustainable use. But even I sometimes feel lost when it comes to the bigger picture. Education needs to connect the dots for people—not just present facts, but explain why it matters to our daily lives and future survival.

I support the idea of fishing bans during breeding seasons and limiting coastal development. Marine policies are improving, but enforcement and public participation still lag. Stronger institutional support and transparent evaluation are essential.

I think the public generally wants to protect the ocean, but their behavior doesn't always match their values. There's still a gap between awareness and action. I've never seen people deliberately pollute, but I have seen carelessness. For example, a plastic bottle left behind by accident, or oil residue from small boats. Minimizing this kind of pollution will require both education and better public services.

What's also important is giving youth more opportunities to participate in marine activities—not just lectures, but hands-on involvement. I once joined a coastal monitoring volunteer team, and it made me feel like I was doing something meaningful.

Participant A14

Gender: Female
Academic Year: Year 2

Excerpt:

I grew up in Shenzhen near Yantian Port, so the ocean has always been a part of my life. It's just a few minutes' walk from my home to the coast. Over the years, I've observed a lot of environmental issues — floating plastic, oil spills, and waste from ships. Even though the city is economically advanced, the marine environment is quite poor. That's why I believe protecting the ocean is really urgent.

The Japanese nuclear wastewater issue also made me think more seriously about marine health. We discussed it in class and wondered how it would affect human health, especially for those who work at sea or rely on seafood. Since I major in shipping, this is directly related to my future. We're often told that the sea provides both livelihood and threat.

I′ve picked up garbage on beaches before. The more urbanized the area, the worse the pollution seems to be. Many of my peers talk about environmental protection, but actual participation is limited. People seem more likely to act if there′s organized activity. I think public institutions should take a stronger role in environmental education.

Marine cultural heritage is also worth protecting. I′ve seen fishing villages that have passed down knowledge and traditions for generations. These communities depend on the sea, and their way of life is disappearing. Protecting that heritage is part of protecting the ocean too.

When it comes to individual responsibility, I think people want to protect the ocean, but often don′t know how. Sometimes the pollution comes from carelessness—like someone fishing and forgetting to pick up their trash. It′s not always intentional. More guidance and civic engagement campaigns might help.

It′s also frustrating that government actions don′t always match their policies. For example, they say they want to protect the ocean, but then approve reclamation projects or allow construction right near fragile coastlines.
