# Supplementary material for: The promotive effect of ocean literacy on marine conservation behavior: A qualitative study based on Chinese university students
Source: PLoS One. 2025 Aug 8;20(8):e0323510. doi: 10.1371/journal.pone.0323510 (PMC12333999; doi:10.1371/journal.pone.0323510)
Supplement: S3_File — (PDF) [file pone.0323510.s003.pdf]

S3_File. Thematic Codebook and Representative Quotes

1、Ocean Literacy

This table summarizes representative anonymized quotes and coded sub-themes related to the overarching theme of Ocean Literacy, drawn from interviews with Chinese university students. The quotes are classified according to five analytical dimensions.

Dimension	Sub-theme (Node)	Participant	Representative Quote (Anonymized)	Interpretation (Optional)
Public Knowledge of the Ocean	Media-based knowledge acquisition	A1	"Marine knowledge is often acquired passively, such as when I see news articles, videos, or course content."	Indicates reliance on indirect or incidental knowledge sources.
Cognition about the Ocean	Misunderstandings or myths	A11	"Some of my friends think the ocean is endless and cannot be polluted, which is obviously not true."	Highlights public misconceptions and ecological illiteracy.
Individual Ocean Literacy Levels	Awareness gaps among peers	A7	"When I asked classmates about ocean issues, many just shrugged. They didn't know much."	Suggests uneven ocean literacy among students.
Role and Significance of Ocean Literacy	Cultural integration	A6	"In my hometown, the sea is not just nature—it's part of our festivals and songs."	Demonstrates how ocean literacy is embedded in local cultural identity.
Components of Ocean Literacy	Empathy and ethical concern	A8	"Sometimes I feel sad when I see videos of turtles trapped in plastic. It makes me think we should do something."	Highlights the emotional and moral foundation of environmental action.

2、Marine Responsibility and Behavior

This supplementary table presents representative anonymized quotes categorized under the overarching theme of Marine Responsibility and Behavior, derived from interviews with Chinese university students. The quotes reflect personal responsibility, public attitudes, participatory engagement, and practical challenges in marine conservation.

Dimension	Sub-theme (Node)	Participant	Representative Quote (Anonymized)	Interpretation (Optional)
Importance of Marine Responsibility and Conservation Behaviors	Sense of obligation	A1	"I think everyone has a responsibility. We live on this planet, and the ocean is part of our home."	Shows moral framing of marine stewardship.
Public Attitudes and Perceptions Towards Marine Conservation	General support but low engagement	A5	"Most people I know support protecting the ocean, but they don't actually do much about it."	Indicates attitude-behavior gap.
Participation in Marine Activities	Volunteer involvement	A2	"I joined a beach clean-up organized by our school. It made me realize the scale of the issue."	Demonstrates experiential learning through direct engagement.
	Barriers to participation	A7	"I'd like to do more, but there aren't many events near our campus."	Reflects infrastructural and logistical limitations.
Challenges and Strategies in Marine Conservation Actions	Need for collaboration	A6	"Government, media, and schools should work together to promote marine conservation."	Advocates for cross-sectoral cooperation.

3、Marine Policy and Resource Management

This table presents a curated set of anonymized interview quotes related to Marine Policy and Resource Management, derived from Chinese university students. The quotes reflect perceptions of international cooperation, policy skepticism, public awareness of marine resource strain, and governance-related challenges.

Dimension	Sub-theme (Node)	Participant	Representative Quote (Anonymized)	Interpretation (Optional)
Marine Policy and International Cooperation	Policy skepticism	A4	"Even though there are policies, enforcement is weak, especially in remote coastal areas."	Highlights enforcement gap in existing marine policy.
	Government	A9	"I think marine issues should be more of	Suggests need for

	responsibility		a government priority, like climate change.”	stronger policy prioritization.
Public Perceptions of Marine Resource Utilization	Overexploitation awareness	A5	“Too many fishing boats, too few fish—that's what my uncle always says.”	Shows lay awareness of marine resource depletion.
	Conflicting values	A7	“People want development and jobs, but also a clean sea—it's hard to balance.”	Reflects development–conservation dilemma.
Marine Resource Development and Conservation	Sustainable development	A6	“We need to use the ocean wisely, not just take everything now and leave nothing.”	Advocates for long-term sustainable use.
Marine Policy and International Cooperation	Policy skepticism	A4	“Even though there are policies, enforcement is weak, especially in remote coastal areas.”	Highlights enforcement gap in existing marine policy.

4、Human–Ocean Relations

This table presents representative anonymized quotes categorized under the theme of Human–Ocean Relations, including perceptions of the ocean's impact on human life, emotional and attitudinal responses, and personal experiences and interests. These insights are drawn from semi-structured interviews with Chinese university students.

**Dimension**	**Sub-theme (Node)**	**Participant**	**Representative Quote (Anonymized)**	**Interpretation (Optional)**
The Impact of the Ocean on Humans	Marine economy and livelihoods	A3	“My uncle is a fisherman. What happens at sea affects our whole family's income.”	Highlights dependence on ocean-based economies.
	Ecological and health connection	A14	“If the sea is polluted, it affects our water and health—especially in coastal areas where people use desalinated water.”	Links ocean condition to everyday human health.
Emotions and Attitudes Toward the Ocean	Emotional healing	A7	“When I'm upset, I go to the beach. Just sitting there makes me feel calm.”	Reflects the therapeutic function of marine environments.
	Ambivalence	A13	“I like the ocean, but I'm also a bit	Captures

	(love and fear)		scared of it. It's too vast and mysterious."	ambivalent emotional attitudes.
Interest and Experiences Related to the Ocean	Marine recreation and travel	A11	"We once stayed at a seaside hotel. The view was so beautiful—it made me want to go back every year."	Shows positive associations through leisure.
	Educational exposure	A10	"Our class trip to a wetland showed me how trash impacts marine animals. It was shocking."	Illustrates transformative experiential learning.

5、 Marine Environmental Values and Beliefs

This table presents representative anonymized quotes categorized under the theme Marine Environmental Values and Beliefs, based on two analytical dimensions: (1) the composition of marine values and beliefs, and (2) their influence on students' marine-related behaviors. Data are drawn from semi-structured interviews with Chinese university students.

**Dimension**	**Sub-theme (Node)**	**Participant**	**Representative Quote (Anonymized)**	**Interpretation (Optional)**
Composition of Marine Values and Beliefs	Human–nature coexistence	A14	"When building offshore projects, we should protect mangroves and marine habitats. Respecting nature and coexisting with it is key."	Highlights a harmony-based environmental belief.
	Value type reflection	A6	"Some people are egoistic, others altruistic. I think I lean toward protecting nature—that's altruistic."	Demonstrates internal reflection on environmental value orientation.
Impact of Marine Values and Beliefs	Motivation for conservation	A13	"Once you have those beliefs, you start holding yourself back—like when you see someone polluting, you feel you should stop it."	Shows moral norms guiding environmental actions.
	Generational and social influence	A10	"Values and beliefs shape how we behave. They also shape how our generation thinks about protecting the ocean."	Suggests values as long-term drivers of social change.

6、Marine Environmental Issues

This table presents a condensed selection of representative anonymized quotes categorized under the theme Marine Environmental Issues, across three dimensions: (1) societal response to marine

pollution, (2) public impact of nuclear wastewater incidents, and (3) perceived impact of marine environmental issues.

Dimension	Sub-theme (Node)	Participant	Representative Quote (Anonymized)	Interpretation (Optional)
Marine Environmental Issues	Societal response to marine pollution	A3	"When there's an oil spill, I feel really angry. Why do people just dump waste into the sea?"	Indicates moral concern and frustration with irresponsible practices.
	Public impact of nuclear wastewater incidents	A6	"I saw the news about Japan's nuclear wastewater. I felt afraid. What if it affects our seafood?"	Reflects fear and perceived personal risk.
	Impact of marine environmental issues	A1	"Plastic and trash are everywhere. The ocean is becoming a giant garbage dump."	Highlights widespread environmental degradation.
		A4	"Sometimes I wonder if future generations will still be able to swim in the sea."	Expresses concern about intergenerational environmental loss.

7、Marine Society and Culture

This table presents representative anonymized quotes drawn from semi-structured interviews with Chinese university students, classified under two analytical dimensions: (1) importance of marine society and culture, and (2) protection and inheritance of marine cultural heritage.

Dimension	Sub-theme (Node)	Participant	Representative Quote (Anonymized)	Interpretation (Optional)
Marine Society and Culture	Importance of marine society and culture	A6	"I think people living by the sea naturally have a connection with the ocean, like their festivals and local customs."	Shows how coastal traditions are deeply tied to the marine environment.
	Protection and inheritance of marine cultural	A13	"We should record and protect fishing techniques and sea legends before they vanish with the older generation."	Emphasizes the need to document and preserve

	heritage			intangible marine heritage.
		A7	"Schools can include marine stories and traditional practices to help us remember and respect our ancestors' way of life."	Suggests integrating marine heritage into education for cultural continuity.

8、Marine Education and Awareness

This table presents a concise set of anonymized quotes from semi-structured interviews with Chinese university students, categorized under four key sub-themes related to marine education and awareness.

**Dimension**	**Sub-theme (Node)**	**Participant**	**Representative Quote (Anonymized)**	**Interpretation (Optional)**
Marine Education and Awareness	Current state of marine education	A2	"We barely have marine-related courses in our university unless you're in a specialized program."	Points to lack of integration in general education curricula.
	Approaches to marine popular science education	A6	"Short videos and online campaigns are more attractive to young people—they should use those more."	Suggests media-based strategies for better youth engagement.
	The role and significance of marine education	A10	"I didn't realize how serious marine pollution was until I attended a marine protection event in school."	Highlights transformative power of experiential education.
